# rbcL-based dataset on intra-specific diversity and conservation of *Adansonia digitata* L. (Malvaceae) in the savannah belt of Nigeria

**DOI:** 10.1016/j.dib.2023.109129

**Published:** 2023-04-07

**Authors:** Conrad Asotie Omonhinmin, Panrot Peace Piwuna, Miracle Omolara Aderanti, Abimbola Odunayo Bolade

**Affiliations:** Department of Biological Sciences Biotechnology Cluster, College of Science and Technology, Covenant University, Canaan land Ota, Ogun State, Nigeria

**Keywords:** *Adansonia digitata*, Baobab, Genetic diversity, Evolution, Conservation, Phylogeny, rbcL gene

## Abstract

The African baobab species belong to the family Malvaceae and the genus *Adansonia*. The disjointed tree thrives in arid or semi-arid regions, is native to the thorn woodlands of Africa, along tracks, and is associated with human-populated forest areas. It is considered indigenous to Central and West Africa and has been introduced to the Arabian Peninsula, South-East Asia, the Indian sub-continent, and the Caribbeans. *Adansonia digitata* is a multifunctional tree with a long lifespan of over 1000 years old. The leaves, roots, flowers, fruit pulp, seeds and barks are used for food, medicine, or other ethnic-practices. The utilisation level and distribution are significantly undermined by climate change and poor use practices. The data set offers insight into the distribution pattern and genetic diversity of *Adansonia digitata* across the savannah belt of Nigeria using the rbcL gene.

**Specifications Table**

**Table:** 

Subject	Biological Sciences
Specific subject area	Agricultural, Genetic diversity, Molecular Phylogenetics, Evolution
Type of data	Tables, Figure, Repository data
How the data were acquired	Amplification of the rbcL gene through PCR and DNA Sanger sequencing.
Data format	Raw, Analyzed
Description of data collection	Leaf samples of *Adansonia digitata* were collected across the Savannah belt of Nigeria, Silica gel dried, and preserved under -80oC (Table 1). All accessions were evaluated using rbcL primers. The population diversity, nucleotide and amino acid compositions of the accessions were estimated using DnaSP 4.5. Codon usage bias and the codon usage indices were estimated using CodonW.
Data source location	The data locations are summarised in: Table 1, Table 2, Table 3, Table 4, Table 5 and the NCBI GenBank.
Data accessibility	The sequence data of the accessions have been deposited in NCBI GenBank database sequence and has the following accession numbers; MT302823.1, MT302822.1, MT302821.1, MT302820.1, MT302819.1, MT302818.1, MT302817.1, MT302816.1, MT302815.1, MT302814.1, MT302813.1, MT302812.1, MT302811.1, MT302810.1, MT302809.1, MT302808.1, MT302807.1, MT302806.1, MT302805.1, MT302804.1, MT302803.1, MT302802.1, MT302801.1, MT302800.1, MT302799.1, MT302798.1, MT302797.1, MT302796.1, MT302795.1, MT302794.1, MT302793.1, MT302792.1, MT302791.1, MT302790.1, MT302789.1, MT302788.1, MT302787.1, MT302786.1, MT302785.1 https://www.ncbi.nlm.nih.gov/nuccore/?term=Adansonia±digitata±Omonhinmin[1].

## Value of the Data

• The data provides information on the distribution and genetic diversity of *Adansonia digitata* sequences across the Savannah belt of Nigeria using information from partial rbcL gene sequences, nucleotide polymorphism and amino acid composition.
• The data identifies areas of high genetic diversity of *Adansonia digitata,* which can be adopted for the improvement of the species and germplasm bank for species conservation.
• The rbcL gene sequences can be employed by plant molecular taxonomists to trace the molecular phylogeny, evolution as well as sub-speciation of *Adansonia digitata*.
• This data presents information on the species' amino acid composition and codon usage bias.

## 1 Data Description

**Fig. 1:**
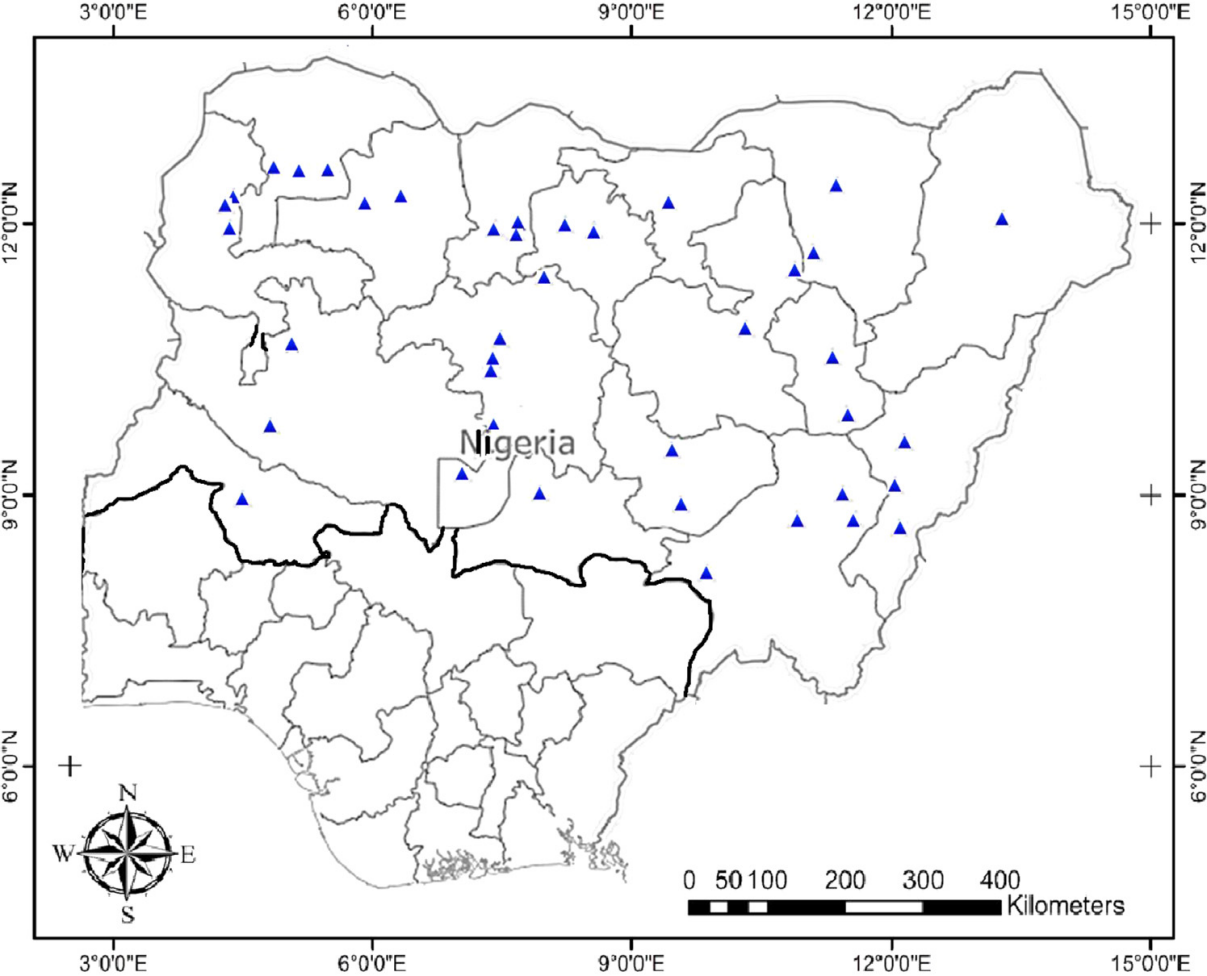
Savannah belt and species range in Nigeria with collection sites.

The study presents the nucleotide sequences of rbcL gene for the 39 *A. digitata* accessions deposited on NBCI GenBank. Fig. 1; presents the map of the collection sites across Guinea, Sudan and Sahel Savanna of the study area [2], [3], [4], [5], [6]. Table 1; lists the accessions studied (39), site collection details and NCBI GenBank accession numbers. Table 2; presents the state-wise (within-species) genetic diversity of *A. digitata*, the number of segregating sites (S), within-group mean distance, nucleotide diversity (π), nucleotide diversity (SD), and average no of nucleotide differences (k). Table 3; presents the genetic diversity of *A. digitata* amongst the accessions; Table 4; highlights the percentage nucleotide composition (TCAG) of *A. digitata* accessions. Table 5; records the amino acid composition of nucleotides *of A. digitata* accessions.

**Table 1:** NCBI s and site collection information for 39 *A. digitata* for the study.

SN	GenBank Accession Number (ф)	Herbarium number (vouchers)	State	Site	Altitude (m)	Latitude NS	Longitude EW
1	MT302785	NANO129	Nasarawa	Kokona	316	8°55′31.0"N	7°57′07.4"E
2	MT302786	ADNO61	Adamawa	Danaba	501	8°29′22.3"N	12°01′17.1"E
3	MT302787	ADNO69	Adamawa	Mayo Farang	358	8°57′07.2"N	11°59′02.4"E
4	MT302788	ADNO71	Adamawa	Dwam	146	9°27′57.6"N	12°04′51.6"E
5	MT302789	BANO118	Bauchi	Kubi	502	10°45′57.6"N	10°17′16.8"E
6	MT302790	GMNO78	Gombe	Tula Wange	492	9°47′13.2"N	11°25′12.0"E
7	MT302791	GMNO83	Gombe	Gadam	566	10°26′49.2"N	11°02′27.6"E
8	MT302792	KANO143	Kaduna	Gwagwada	648	10°16′15.6"N	7°24′07.2"E
9	MT302793	KANO152	Kaduna	Rumi	626	11°21′46.8"N	7°58′58.8"E
10	MT302794	KANO126	Jigawa	Chaichai	390	12°04′34.8"N	9°19′02.9"E
11	MT302795	KANO139	Kaduna	Iddah	535	9°21′57.6"N	7°18′03.6"E
12	MT302796	KANO144	Kaduna	Kakau,	615	10°24′03.6"N	7°24′03.6"E
13	MT302797	KANO148	Kaduna	Regachukun	662	10°38′38.4"N	7°29′09.6"E
14	MT302798	KENO208	Kebbi	Aliero S Fafa	267	12°16′15.6"N	4°27′10.8"E
15	MT302799	KENO213	Kebbi	Kimba,	202	12°10′40.8"N	4°22′19.2"E
16	MT302800	KENO218	Kebbi	Sarandosa,	252	11°55′26.4"N	4°25′37.2"E
17	MT302801	KNNO163	Kano	Unguwar Rimi	492	11°54′28.8"N	8°32′42.0"E
18	MT302802	KNNO167	Kano	Rimin Gado	536	11°57′57.6"N	8°14′06.0"E
19	MT302803	KTNO168	Katsina	Dayi	622	11°57′43.2"N	7°42′43.2"E
20	MT302804	KTNO175	Katsina	Yamama	600	11°52′15.6"N	7°39′25.2"E
21	MT302805	KTNO178	Katsina	Yagoje	604	11°54′46.8"N	7°25′37.2"E
22	MT302806	KWNO276	Kwara	Pakunmo	293	8°47′42.0"N	4°33′07.2"E
23	MT302807	NINO222	Niger	Ibbi	212	9°38′13.4"N	4°54′21.4"E
24	MT302808	NINO258	Niger	Auna East	238	10°15′01.0"N	4°58′59.5"E
25	MT302809	PINO123	Plateau	Shimankar	159	8°36′54.0"N	9°30′46.8"E
26	MT302810	SONO200	Sokoto	Lamba Tureta	282	12°39′28.8"N	5°34′51.6"E
27	MT302811	SONO202	Sokoto	Kambama	263	12°36′46.8"N	5°12′21.6"E
28	MT302812	SONO205	Sokoto	Shagari	281	12°37′15.6"N	4°59′24.0"E
29	MT302813	TANO39	Plateau	Kabwir Pada	741	9°24′51.6"N	9°35′09.5"E
30	MT302814	TANO40	FCT	Ibwa	201	9°05′45.6"N	7°04′15.6"E
31	MT302815	TANO46	Taraba	Gunduma	188	8°32′56.4"N	10°51′00.0"E
32	MT302816	TANO51	Taraba	Bali-Jalingo	221	8°50′42.0"N	11°22′51.6"E
33	MT302817	TANO56	Taraba	Zongon Kombi	204	8°51′32.8"N	11°05′15.9"E
34	MT302818	TANO57	Taraba	Sankani	259	8°41′11.1"N	11°16′21.4"E
35	MT302819	TANO58	Yobe	Gudugurka	350	12°16′56.8"N	11°11′17.9"E
36	MT302820	YONO106	Yobe	Zubo	403	11°38′06.0"N	10°02′27.6"E
37	MT302821	YONO110	Bornu	Ngubala	333	11°54′06.8"N	12°52′16.1"E
38	MT302822	ZANO187	Zamfara	Dosara Birnin	420	12°27′07.2"N	6°19′22.8"E
39	MT302823	ZANO193	Zamfara	Maru	398	12°20′49.2"N	6°18′50.4"E

**Table 2:** Within-state and intra-specific diversity of *Adansonia digitata* accessions.

State	No. of accessions	No. of segregating sites (S)	Within Group Mean Distance	Nucleotide Diversity(π)	Nucleotide Diversity (SD)	Average no Nucleotide Differences (k)
Adamawa	3	30	0.06	0.05728	0.01639	20.333
Gombe	2	17	0.05	0.05199	0.02599	17
Kaduna	6	51	0.06	0.06125	0.01247	19.6
Kebbi	3	17	0.07	0.05503	0.02182	11.667
Kano	2	41	0.17	0.15018	0.07509	41
Katsina	3	21	0.06	0.05957	0.01749	14
Niger	2	27	0.08	0.07895	0.03947	27
Sokoto	3	30	0.06	0.05970	0.01662	20
Taraba	7	43	0.05	0.04965	0.00957	14
Yobe	2	13	0.05	0.04833	0.02416	13
Zamfara	2	3	0.01	0.00906	0.00453	3

**Table 3:** Genetic diversity amongst the 39 *Adansonia digitata* accessions.

Index	
No. of accessions	39
Number of haplotypes	9
Haplotype diversity	0.448 ± 0.099
Nucleotide Diversity	**0.0136 ± 0.005**
Average No of Nucleotide Difference (k)	0.807
No of segregating sites	11

**Table 4:** Percentage Nucleotide Composition of *Adansonia digitata* accessions.

Accession	T (%)	C (%)	A (%)	G (%)	%GC
MT302785	28.2	23.5	23.8	24.5	48.00
MT302786	29.2	20.5	26.7	23.6	44.10
MT302787	27.2	21.5	26.2	25.1	46.60
MT302788	27.6	22.5	25.9	23.9	46.50
MT302789	29.5	20.7	26.4	23.3	44.00
MT302790	27.5	20.2	28.4	23.9	44.10
MT302791	29.1	21.4	26.3	23.2	44.60
MT302792	29.3	20.2	26.6	23.9	43.10
MT302793	29.5	22.3	25.9	22.3	42.80
MT302794	29.0	19.9	26.9	24.2	44.10
MT302795	29.2	21.1	26.5	23.1	44.60
MT302796	30.4	19.7	26.4	23.4	44.10
MT302797	29.7	19.6	27.5	23.2	44.20
MT302798	25.9	22.9	29.0	22.2	45.00
MT302799	29.4	19.9	28.0	22.7	42.70
MT302800	31.2	20.2	26.3	22.3	42.50
MT302801	28.7	21.6	26.5	23.2	44.80
MT302802	30.0	20.8	26.8	22.5	43.30
MT302803	25.1	22.7	26.2	26.0	48.70
MT302804	29.9	20.4	26.8	22.8	43.20
MT302805	27.9	19.7	28.2	24.2	43.90
MT302806	29.3	20.2	26.9	23.7	43.80
MT302807	28.9	20.1	26.1	24.9	45.00
MT302808	29.5	20.8	26.0	23.7	44.40
MT302809	28.4	21.0	27.1	23.5	44.50
MT302810	30.4	19.4	26.6	23.6	43.00
MT302811	28.8	20.6	27.0	23.7	44.30
MT302812	29.8	20.6	27.4	22.2	42.80
MT302813	29.3	20.2	26.9	23.7	43.90
MT302814	27.8	20.1	27.6	24.5	44.60
MT302815	27.0	21.2	28.1	23.6	44.80
MT302816	29.3	20.1	24.4	26.1	46.30
MT302817	28.0	20.7	28.0	23.2	43.90
MT302818	27.9	20.6	27.6	23.9	44.50
MT302819	29.1	20.1	27.1	23.8	43.90
MT302820	28.6	20.3	27.3	23.8	44.10
MT302821	25.7	22.3	26.4	25.7	48.00
MT302822	27.5	20.5	28.4	23.6	44.10
MT302823	29.4	21.0	26.6	22.9	43.90

**Average**	**28.7**	**20.8**	**26.8**	**23.7**	**44.48**

**Table 5:** Amino acid composition of *Adansonia digitata* nucleotides.

Accession	Ala	Cys	Asp	Glu	Phe	Gly	His	Ile	Lys	Leu	Met	Asn	Pro	Gln	Arg	Ser	Thr	Val	Trp	Tyr	Total
MT302785	7.55	1.89	3.77	8.49	4.72	11.32	0.94	4.72	2.83	7.55	0.94	2.83	6.6	1.89	4.72	5.66	9.43	7.55	0.94	5.66	106
MT302786	8.51	1.42	4.26	9.22	4.96	8.51	0.71	3.55	5.67	7.09	0.71	2.13	4.96	1.42	4.96	4.96	9.93	8.51	1.42	7.09	141
MT302787	8.66	1.57	3.94	9.45	6.3	11.81	0.79	3.94	5.51	4.72	0.79	2.36	6.3	1.57	3.94	3.15	10.24	7.09	1.57	6.3	127
MT302788	9.32	1.69	4.24	9.32	1.69	8.47	0	4.24	5.08	5.93	0.85	2.54	8.47	1.69	3.39	5.93	9.32	9.32	1.69	6.78	118
MT302789	7.86	1.43	2.86	10	4.29	7.14	0.71	3.57	4.29	7.14	0.71	2.14	5	2.14	5	5.71	10.71	10	2.14	7.14	140
MT302790	10	1.82	5.45	10.91	3.64	8.18	0.91	2.73	5.45	5.45	0	1.82	5.45	1.82	2.73	3.64	10.91	9.09	1.82	8.18	110
MT302791	11.01	1.83	4.59	11.01	7.34	8.26	0.92	3.67	5.5	5.5	0	0.92	8.26	1.83	1.83	3.67	8.26	7.34	1.83	6.42	109
MT302792	8.8	1.6	4	9.6	5.6	9.6	0.8	3.2	5.6	5.6	0.8	2.4	5.6	1.6	2.4	4	10.4	9.6	1.6	7.2	125
MT302793	10	1.82	4.55	10.91	6.36	8.18	1.82	3.64	5.45	4.55	0	1.82	10	0.91	2.73	4.55	6.36	6.36	1.82	8.18	110
MT302794	9.68	1.61	4.03	94.68	5.65	8.87	0.81	3.23	5.65	4.03	0.81	2.42	5.65	1.61	2.42	4.03	10.48	10.48	1.61	7.26	124
MT302795	9.63	1.48	4.44	8.89	7.41	8.15	0.74	4.44	5.19	8.15	1.48	2.22	6.67	1.48	4.44	4.44	6.67	6.67	1.48	5.93	135
MT302796	9.02	1.5	3.76	9.77	6.77	9.02	0.75	3.76	4.51	8.27	0.75	2.26	3.76	1.5	5.26	7.52	8.27	6.77	0.75	6.02	133
MT302797	9.02	1.64	4.1	9.02	5.74	9.02	0.82	4.1	5.74	3.28	0.82	3.28	5.74	1.64	2.46	4.92	9.84	9.84	1.64	7.38	122
MT302798	5.95	1.08	3.24	7.57	4.86	9.19	0.54	3.78	7.57	6.49	0.54	3.24	9.19	3.24	4.86	3.78	9.73	7.57	2.16	5.41	185
MT302799	7.14	1.43	4.29	10	4.29	7.86	0.71	3.57	5	7.14	0.71	2.86	5.71	1.43	5	7.14	9.29	7.86	1.43	7.14	140
MT302800	4.88	3.66	3.66	7.32	4.88	7.32	2.44	6.1	3.66	9.76	1.22	3.66	2.44	3.66	7.32	8.54	4.88	7.32	0	7.32	82
MT302801	11.65	1.94	4.85	10.68	8.74	6.8	0.97	1.94	5.83	5.83	0	2.91	7.77	0.97	3.88	3.88	6.8	6.8	1.94	5.83	103
MT302802	7.34	1.69	4.52	7.34	3.39	9.6	0.56	3.95	5.65	9.6	0	3.39	7.34	2.26	5.08	5.65	7.34	6.78	1.13	7.34	177
MT302803	7.33	2.67	2	9.33	3.33	12.67	0.67	3.33	5.33	9.33	0.67	4.67	8	2	6	4.67	6.67	4.67	1.33	5.33	150
MT302804	7.91	1.44	3.6	10.07	4.32	8.63	1.44	5.76	5.04	7.19	0.72	2.16	6.47	0.72	3.6	4.32	9.35	8.63	1.44	7.19	139
MT302805	9.32	1.69	3.39	11.02	4.24	9.32	1.69	4.24	5.93	6.78	0	1.69	4.24	1.69	2.54	4.24	10.17	8.47	1.69	7.63	118
MT302806	9.68	1.61	4.03	9.68	5.65	8.87	0.81	3.23	5.65	4.84	0.81	2.42	5.65	1.61	2.42	4.03	10.48	9.68	1.61	7.26	124
MT302807	10.45	1.49	4.48	10.45	5.22	8.96	0.75	3.73	5.97	8.21	0.75	2.24	2.99	0.75	4.48	6.72	7.46	8.21	0.75	5.97	134
MT302808	7.89	1.75	4.39	10.53	6.14	8.77	0.88	3.51	5.26	5.26	0.88	1.75	7.02	1.75	2.63	4.39	11.4	7.89	1.75	6.14	114
MT302809	10.09	1.83	4.59	11.01	4.59	8.26	0.92	2.75	5.5	5.5	0	0.92	6.42	1.83	2.75	4.59	10.09	9.17	1.83	7.34	109
MT302810	8.11	1.8	4.5	9.91	7.21	9.91	0.9	1.8	5.41	4.5	0.9	1.8	6.31	1.8	2.7	3.6	10.81	8.11	1.8	8.11	111
MT302811	9.92	2.29	3.82	9.92	3.82	7.63	0.76	4.58	4.58	6.87	0.76	2.29	5.34	1.53	3.82	3.82	9.92	9.16	1.53	7.63	131
MT302812	8.94	1.63	3.25	12.2	8.13	8.13	0.81	3.25	5.69	4.88	0.81	1.63	7.32	1.63	2.44	4.88	9.76	7.32	0.81	6.5	123
MT302813	8.8	1.6	4	9.6	5.6	8.8	0.8	3.2	5.6	5.6	0.8	2.4	5.6	1.6	2.4	4.8	10.4	9.6	1.6	7.2	125
MT302814	7.95	1.99	4.64	9.27	3.97	9.27	0.66	4.64	5.96	6.62	0.66	1.99	3.97	2.65	3.97	6.62	9.93	7.28	1.99	5.96	151
MT302815	6.78	2.26	3.39	9.04	4.52	9.6	0.56	3.95	6.21	7.34	0.56	2.82	6.78	3.39	4.52	4.52	9.6	6.21	1.69	6.21	177
MT302816	9.57	2.13	4.26	9.57	6.38	10.64	1.06	2.13	5.32	6.38	0	1.06	6.38	2.13	3.19	3.19	9.57	8.51	2.13	6.38	94
MT302817	10.09	1.83	3.67	11.01	4.59	8.26	0.92	3.67	6.42	5.5	0	0.92	5.5	1.83	2.75	4.59	11.01	8.26	1.83	7.34	109
MT302818	10.91	1.82	4.55	10.91	3.64	8.18	0.91	2.73	5.45	5.45	0	1.82	5.45	1.82	2.73	3.64	10.91	9.09	1.82	8.18	110
MT302819	8.27	1.5	4.51	9.02	5.26	9.77	0.75	3.01	6.02	7.52	0.75	2.26	4.51	1.5	3.76	6.02	9.02	8.27	1.5	6.77	133
MT302820	8.13	1.63	3.25	10.57	5.69	9.76	0.81	4.88	4.88	4.88	0.81	2.44	6.5	2.44	2.44	3.25	10.57	8.94	1.63	6.5	123
MT302821	11.24	1.12	4.49	11.24	3.37	8.99	1.12	2.25	6.74	4.49	0	0	7.87	1.12	3.37	3.37	11.24	8.99	2.25	6.74	89
MT302822	10	1.82	4.55	10.91	3.64	8.18	1.82	2.73	5.45	5.45	0	1.82	5.45	1.82	2.73	3.64	10.91	9.09	1.82	8.18	110
MT302823	9.15	1.41	4.23	9.15	4.93	7.75	0.7	4.23	5.63	7.04	0.7	2.11	5.63	1.41	4.23	4.23	10.56	8.45	1.41	7.04	142

Average	8.77	1.73	4.02	9.75	5.1	8.95	0.88	3.67	5.49	6.4	0.57	2.28	6.14	1.82	3.71	4.75	9.44	8.12	1.57	6.83	125.7

## 2 Experimental Design, Materials and Methods

### 2.1 Plant Material

*Adansonia digitata* accessions (39) were collected during a nationwide expedition across 17 states and Nigeria's Federal Capital City (FCT) in 2016-2017 (Fig. 1). The fresh leaf samples of the accessions were silica gel dried, packed in labelled air-tight bags and held at −80°C prior to molecular analysis at the Bioscience Laboratory, International Institute of Tropical Agriculture (IITA), Ibadan Nigeria.

### 2.2 Genomic DNA Extraction

Genomic DNA was extracted using the CTAB protocol [7], and quality and quantity were authenticated using the ThermoFischer® Nanodrop spectrophotometer ND-8000-GL

### 2.3 Gene Amplification and DNA Sequencing

The PCR amplicons were sequenced at the International Institute of Tropical Agriculture (IITA), Ibadan, Nigeria.

Using the rbcL primers H1f - forward (CCACAAACAGAGACTAAAGC) and Fofana - Reverse (GTAAAATCAAGTCCACCGCG) [8]; a portion of the species chloroplast ribulose 1, 5-bisphosphate carboxylase (rbcL) gene was amplified.

25 µL PCR cocktail mix was generated for each sample with the following 2.5 µL of 10x PCR buffer, 1.0 µL of 50 mM MgCl2, 1.5 µL of 5 pMol forward primer, 1.5 µL of 5 pMol reverse primer, 1.0 µL of DMSO (Dimethyl sulfoxide), 2.0 µL of 25 mM dNTPs, 0.06 µL of Taq, 2.0 µL of 100 ng/µL DNA (extract), and 13.44 µL of H_2_0.

PCR amplification of the DNA samples was performed on GeneAmp™ PCR System 9700  thermocycler; with initial denaturation at 94°C, 180 sec, 10 cycles; denaturation at 94°C, 15 sec; annealing at 65°C, 30 sec; extension at 72°C, 90 sec, 10 cycles; denaturation at 93°C, 15 sec; annealing at 55°C, 30 sec; extension at 72°C for 90 sec, 30 cycles; and final extension at 72°C, 300 sec. Amplicons were held at 10°C until use.

### 2.4 Visualisation of AMPLICONS

Amplified products were loaded on 1.5% agarose gel with 1kbplus GeneRuler DNA Ladders (Thermo Fisher Scientific®). Gel components ran at 100 volts for 1 hr. The resultant bands were visualised under a UV light trans-illuminator.

### 2.5 Editing and Evaluation of DNA Sequences and Submission

De novo (unrevised) forward and reverse reads assembly were performed to generate consensus sequences using the Geneious assembler (GENEIOUS Prime 2020.1). Where assembly of De novo was not feasible, the NCBI database mapped forward and reverse reads to the previously submitted rbcL gene sequence (GU981721.1). The reference sequence was trimmed using the sequencing primers before mapping the sequencing reads to the guide. Using the Geneious alignment tool, generated sequences were individually aligned with the reference rbcL sequence after assembly to check for sequencing errors that might result in translation errors. Cleaned lines were then submitted to GenBank using Bankit.

### 2.6 Data Analysis

Generated *A. digitata* accession sequences were aligned to generate consensus sequences using Geneious Basic [9]. The default settings were employed for the consensus alignment to obtain the % GC and sequence lengths. The rbcL sequences for *A. digitata* ranged from 245-555 bp for the study.

Population diversity indices were assessed using DnaSP 4.5 [10]. The indices analysed include haplotype number (h), haplotype diversity (Hd), numbers of segregating sites (S), nucleotide diversity (π) and average number of pairwise nucleotide differences within the population (K).

The codon usage frequency, amino acid and nucleotide compositions of each *Adansonia digitata* accessions sequences were determined using DnaSP 4.5. Codon usage indices were estimated using CodonW on Galaxy server (https://galaxy.pasteur.fr/).

## Ethics Statements

The field data presented in Table 1, were obtained via open field collection visits, and did not require informed consent. No part of the data was obtained from any Social Media platform.

## CRediT Author Statement

**Conrad Asotie Omonhinmin:** Conceptualization, Funding acquisition, Methodology, Supervision, Writing – review & editing; **Panrot Peace Piwuna:** Investigation, Writing – original draft; **Miracle Omolara Aderanti:** Investigation, Formal analysis, Writing – review & editing; **Abimbola Odunayo Bolade:** Investigation, Data Curation.

## Declaration of Competing Interest

The authors declare that they have no known competing financial interests or personal relationships that could have appeared to influence the work reported in this paper.

## Data Availability

rbcL-based dataset on intra-specific diversity and conservation of Adansonia digitata L. (Malvaceae) in the savannah belt of Nigeria. (Original data) (NBCI GenBank).
